# The “more at home with dementia” program: a randomized controlled study protocol to determine how caregiver training affects the well-being of patients and caregivers

**DOI:** 10.1186/s12877-018-0948-3

**Published:** 2018-10-22

**Authors:** Elizabeth G. Birkenhäger-Gillesse, Boudewijn J. Kollen, Sytse U. Zuidema, Wilco P. Achterberg

**Affiliations:** 1 Department of General Practice and Elderly Care Medicine, University Medical Center Groningen, Hanzeplein 1, 9713 GZ Groningen, the Netherlands; 2 Laurens Care Centers, Nieuwe Binnenweg 29, 3014 GB Rotterdam, the Netherlands; 3 Department of Public Health and Primary Care, Leiden University Medical Center, Albinusdreef 2, 2333 ZA Leiden, the Netherlands

**Keywords:** Dementia, Caregiver, Training, Psychosocial intervention

## Abstract

**Background:**

Caring for people with dementia imposes heavy burdens on caregivers, especially spouses. This can lead to depression, anxiety, and physical symptoms in the caregiver, with early institutionalization for the patient. An Australian study reported that a residential caregiver training program delivered in medical settings could delay nursing home admission, lower mortality, reduce psychological morbidity in caregivers, and lower healthcare costs. In this replication study, we aim to determine the effectiveness of an adaptation of this program to non-medical settings in the Dutch health care system.

**Methods:**

A randomized controlled study design will be used, comparing an intervention group with a control group. The intervention will last for five days and will be delivered in either a holiday park or a bed and breakfast setting. The control group will receive care as usual. Data will be collected at baseline and after 3 and 6 months, and outcomes will be assessed in the caregiver group and in the dementia group. The primary outcome will be caregiver-related quality of life after 3 months. The main secondary outcome will be the neuropsychiatric symptoms in the dementia group. Secondary outcomes in the dementia group will be activities of daily living and instrumental activities of daily living, use of health facilities, quality of life, agitation, dementia severity, and use of psychotropic medication. Secondary outcomes in the caregiver group will be the subjective and objective burdens, health and health care facility use, psychotropic medication use, depression, anxiety, and perseverance time.

**Discussion:**

We anticipate that the outcomes will allow us to confirm the effectiveness of the intervention, and in turn, potentially inform the introduction of this program into care plans. It is also expected that the experiences and recommendations of participants will help us to develop the training program further.

**Trial registration:**

Registered in the Netherlands Trial Register on March 9, 2016, number 5775.

## Background

People with dementia prefer to live at home for as long as possible. In the Netherlands, it is estimated that 70% of people with dementia currently live in the community [1]. Many of these require 24-h support and care from a next of kin, typically a spouse, child, or other relative. Given that the number of people with dementia is expected to double over the next 20 years [2], growing numbers of relatives will be expected to provide care for a relative with dementia. However, caregivers themselves can experience high levels of burden and stress as a result of this role, ultimately leading to mental illness [3, 4] or early institutionalization of the dementia sufferer.

A prospective cohort study reported that half of all caregivers experienced excessive burden that increased over time and led to aggression, depression, anxiety, and poorer physical health [5]. Evidence also suggests that caregivers are at increased risk of serious illnesses and death [6]. Research on the effect of long-term caregiving has shown that it has long-term consequences. Indeed, in one study, psychological well-being among caregivers did not improve to levels comparable with those in non-caregivers after the person they cared for died or was admitted to a nursing home [7]. The high levels of burden to which a caregiver is exposed, typically through the poorer cognition and neuropsychiatric symptoms of the person with dementia [8], can also increase the risk of the care recipient being placed in a nursing home. Psychological morbidity in caregivers is also a predictor of mortality for people with dementia [9].

Developing effective interventions that target caregivers can benefit both the caregiver themselves and the person for whom they care. There is extensive literature on this issue, but heterogeneity of interventions and outcomes make it difficult to identify the most effective and relevant interventions. Most reviews have only indicated mild to moderate effect sizes with common interventions, while some subgroup analyses have shown stronger positive effects [10–21]. Interventions in these subgroups have tended to incorporate the following: group training, social support, cognitive interventions, intensive programs, multi-model programs with multiple components, programs that focus on the caregiver/patient dyad, caregiver training, caregiver family involvement, adapting to caregivers’ needs, and providing psychological education. By contrast, reviews on the effects of respite care have shown that this intervention generates no benefits [22, 23]. In Australia, a residential caregiver training program has been developed that incorporates most of the components shown to be effective in these reviews. This showed promise, effectively delaying nursing home admission, lowering mortality, reducing psychological morbidity in caregivers, and lowering care costs [9, 24–26]. This program is now being investigated in another study (entitled the “Going to Stay at Home” program), for which a protocol was recently published [27].

In the Netherlands, dementia is typically diagnosed in geriatric outpatient clinics, by general practitioners (GPs), or by elderly care physicians. Following diagnosis, patients are referred to a case manager specializing in dementia, who supports the caregiver and gives advice concerning home care, day care, and nursing home admission. However, there are significant local differences regarding the availability of and the accessibility to these health services. Moreover, the Australian interventions were delivered in nursing home or hospital settings, and we thought it would be interesting to known whether such services can be delivered in non-medical residential settings.

In this study, we will adapt a caregiver training program for use in informal non-medical settings in the Dutch health care system and determine the effectiveness of that program based on several key outcomes. The primary outcome will be the caregiver-related quality of life after 3 months. The secondary outcomes will be two-fold. First, for the caregiver, we will assess the quality of life course, caregiver burden, health, care utilization, psychotropic medication use, depressive and anxiety symptoms, and perseverance time. Second, for people with dementia, we will assess the courses of neuropsychiatric symptoms, activities of daily living, utilization of care, costs of care, quality of life, agitation, use of psychotropic medication, and severity of dementia.

## Methods

### Study design

A randomized controlled trial study design will be used (registered at the Dutch Trial Register; Trial ID, NTR5775). After providing informed consent, participant dyads (caregiver and a person with dementia) will be randomly assigned to an intervention or a control group. In the intervention group, participant dyads will take part in the training program (entitled “More at Home with Dementia”), while in the control group, participants will receive care as usual. Data will then be collected at three points: at baseline and during meetings at 3 and 6 months after the intervention. Additionally, information on the use of care and health facilities will be collected by telephone after 6 and 18 weeks. People who drop out of the intervention or control group will be contacted at regular times by phone to request information on the date of admission to a nursing home or death, as applicable.

### Participants

Participants will be recruited by either professional referral or self-referral. The project will be promoted among geriatricians, case managers, day care centers, mental health institutions for the elderly, and GPs, who will be asked to refer appropriate participants. To arouse interest and promote participation in the general public, the study will be advertised in a radio interview, in newspaper advertorials, on social media (e.g., Facebook), and on the website of the Dutch Alzheimer Association (*Alzheimer Nederland*). These sources will direct potential participants to a dedicated website (http://beterthuismetdementie.laurens.nl/) and a phone number, from where detailed information can be gained and participation can be sought.

### Inclusion and exclusion criteria

We will include people with dementia if they meet the following criteria: (1) they have a confirmed diagnosis of dementia according to Diagnostic and Statistical Manual of Mental Disorders; (2) they live at home with their primary caregiver; and (3) they are able to understand and communicate in Dutch. We will exclude those who refuse to participate (by either verbal or behavioral indication) and who show aggressive or wandering behaviors. The only inclusion criterion for caregivers will be that they are able to understand and communicate in Dutch.

### Randomization

Application will be by returning a signed informed consent form. Then, participant dyads (caregiver and patient) will be randomly assigned to either the intervention group or the control group by means of block randomization. The randomization sequence will be recorded in a separate document that will be unavailable to the research assistant responsible for participant enrolment.

### Intervention

We will use the course materials and facilitator’s guide of the “Going to Stay at Home” program, both for the structure and for the content of the sessions. The content will be adapted only when necessary, such as when it is not considered applicable to our setting.

#### Setting

The intervention will last for a total of five days and will take place at either a holiday park (https://www.zuytlandbuiten.com/) or a bed and breakfast (https://www.deappelgaard-gouderak.nl/) near Rotterdam. Accommodation will be provided to host six couples, each with their own bedroom. Groups will then consist of three to six participant dyads.

#### Program for caregivers

The caregivers will attend 14 psycho-educational sessions. These will be delivered in an informal setting by a psychologist, a physiotherapist, an occupational therapist, an elderly care physician, a speech therapist, a dietician, and a social worker. Sessions will include didactic elements, group work, modeling, and role play, as follows:

1. *Combating social isolation.* Participants will be stimulated to share their experiences and to explore available social contacts, support and needs. An open and supportive relationship can be established between caregivers during the intervention and subsequent meetings.
2. *Medical aspects of dementia.* Relevant information will be provided about the types of dementia and associated changes in behavior, as well as accompanying symptoms like apraxia and aphasia. Frequently occurring complications like delirium and depression will be explained.
3. *Planning for the future.* The need to plan for future emergencies and unforeseen events will be discussed, including driving, legal and financial matters, and advance care directives. The issues will be explored during the sessions, but participants will be advised to explore them further at home, preferably with their partner, and if necessary with the help of relatives or professionals.
4. *Re-rolling.* This session will explore issues regarding changes in the roles and responsibilities of the caregiver and the person with dementia. Caregivers will be assisted in considering ways to take over tasks from the people with dementia while preserving their dignity. Attention will then be paid to how to deal with new responsibilities.
5. *Reminiscence and orientation.* Caregivers will be provided with information on techniques that encourage reminiscence (e.g., life history book, music, objects representing notable events) and orientation to the environment and reality (e.g., signs and clocks designed for people with dementia).
6. *Communication.* Information will be given about aphasia and how it affects communication, together with management strategies. Attention will be paid to problems with swallowing.
7. *Assertion.* It will be important to provide training about assertive, non-assertive, and aggressive behaviors, and to provide strategies to cope with criticism. The rights of caregivers should be emphasized and observed, and their needs should be heard and met.
8. *Therapeutic use of activities.* We will identify meaningful and enjoyable activities that are increasingly difficult for the people with dementia to perform. Caregivers will be taught a method of activity analysis that will enable them to break these activities down into component steps and modify or eliminate steps that cannot be completed.
9. *Organization of work and safety in the home.* This session will focus on simplifying, prioritizing, and using outside assistance to achieve a better balance between work and leisure. Attention will be paid to safety at home.
10. *Nursing skills.* Issues associated with dementia will be discussed, such as incontinence, personal care, medication, and mobility. Caregivers will be educated about how to give personal care to a nursing standard, focusing on washing, assistance with dressing, assistance with rising from a chair or bed, and managing incontinence.
11. *Fitness.* The benefits of exercise for both the caregiver and the person with dementia will be emphasized. If possible, exercises or walks will be included in the program. In case of disability, alternatives to walking will be discussed.
12. *Nutrition.* Attention will be paid to the changes in diet, food intake, food preferences, and nutritional needs of people with dementia. Caregivers will be given advice about how to deal with these issues and about how to improve the eating experience.
13. *Self-care.* Caregivers will be given the opportunity to identify their support needs. Attention will also be paid to stress management and relaxation techniques.
14. *Using community services.* Information will be distributed about the many support services that are available (e.g., day care and support at home) and about how to access them. Special emphasis will be given to the financial costs of these services for the couple.

#### Program for the people with dementia

The program for the people with dementia will comprise general pleasant activities and sessions focused on the handicaps that come with dementia. General activities will include sessions for relaxation, physical activity, social interaction, and creativity. In the other sessions, we will focus on the handicaps that coincide with dementia, including information on changes caused by dementia (e.g., cognitive) and about how to cope with memory loss by reminiscence. The program will be adjusted according to the abilities and wishes of the group members.

### Outcome measures

The effectiveness of the program will be assessed based on the following primary and secondary outcomes. The primary outcome will be the caregiver-related quality of life after 3 months. The secondary outcomes will be subdivided to those in the caregiver groups and those in the dementia groups. In the caregiver groups, we will assess quality of life, caregiver burden, experienced health, care utilization, psychotropic medication use, depressive and anxiety symptoms, and perseverance time. In the dementia groups, we will assess the courses of neuropsychiatric symptoms, activities of daily living, utilization of care, costs of care, quality of life, agitation, psychotropic medication use, and dementia severity. Secondary outcome variables will be measured at baseline and after 3 and 6 months and compared between the intervention and control groups.

### Instruments

#### Instrument used to assess the primary outcome in caregivers

- *Care-Related Quality of Life-7 dimensions (CarerQol-7D).* This measure scores seven items on care-related satisfaction, relationship problems, mental health, time management, financial problems, social support, and physical health. All items are scored on a scale from no problems to a lot of problems [28]. The scores will be transformed to represent a utility score or tariff between 0 and 100 by adding the relative weights of items [29] (part of The Older Persons and Informal Caregivers Survey Minimum Data Set: TOPICS-MDS)

#### Instruments used to assess the secondary outcomes in caregivers

- *CarerQol - visual analog scale (VAS).* For the VAS, 0 equals completely unhappy and 10 equals completely happy (29) (part of TOPICS-MDS).
- *Self-Rated Burden Scale - VAS.* This is a self-report measure of burden experienced in the caregiver role. Ratings are on a scale from 0 to 10, with higher scores indicating a higher burden (28) (part of TOPICS-MDS).
- *Objective caregiver burden.* This will be graded as the number of hours per week spent on caregiving (part of TOPICS-MDS).
- *RAND-36/short form (SF)-36.* This will be used to measure experienced health or health-related quality of life. The survey includes scales concerning physical functioning, role limitations due to physical health problems, bodily pain, general health perceptions, vitality, social functioning, role limitations due to emotional problems, and general mental health. Higher scores indicate better health [30] (part of TOPICS-MDS).
- *EuroQol-5 Dimensions + Cognition (EQ-5D + C).* This health instrument assesses quality of life on five dimensions: mobility, self-care, activities, pain and discomfort, anxiety and depressed mood, with an additional question about cognitive problems. All items are scored as no problems, some problems, or extreme problems (scored as 1, 2, or 3, respectively) [31, 32] (part of TOPICS-MDS).
- *Psychotropic drug use.* Medication use will be categorized into antipsychotics, anxiolytics, antidepressants, and hypnotics.
- *Center of Epidemiologic Studies-Depression (CES-D).* This instrument screens for depressive symptoms across 20 items. Each item is scored rarely, some or a little of the time, occasionally or a moderate amount of the time, or most or all the time (scored as 0, 1, 2, or 3, respectively). The maximum score is 60, and a score of 16 indicates depression [33].
- *Hospital Anxiety and Depression Scale-anxiety subscale (HADS-A).* This is a well-known 14 item scale that generates ordinal data to determine levels of anxiety or depression (7 items each relate to anxiety and depression). Each item on the questionnaire is scored from 0 to 3, and a person can score from 0 to 21 on the anxiety subscale, with higher scores indicating more symptoms [34].
- *Perseverance Time.* This is defined as how long the caregiver believes that he or she will be able to persevere in their current state provided the situation remains stable. Perseverance time will be categorized as follows, based on earlier research [35, 36]: (1) less than a week; (2) more than a week but less than a month; (3) more than a month but less than six months; (4) more than six months but less than a year; (5) more than a year but less than two years; or (6) more than two years.

#### Instruments used to assess the secondary outcomes in people with dementia

- *The 12-item neuropsychiatric Inventory (NPI).* The main outcome of interest in the dementia group will be the NPI score. This is an informant-rated measure of the frequency and severity of neuropsychiatric symptoms, such as agitation, psychosis, depression and apathy in people with dementia, collected by interview with the caregiver. Higher scores indicate more severe neuropsychiatric symptoms [37].
- *Functional status.* We will assess this with an adapted version of the Katz Index of Independence of Activities for Daily Living (ADLs) and instrumental activities of daily living (IADLs), supplemented with a question concerning mobility (i.e., Katz-15; part of TOPICS-MDS). Caregivers will be asked if assistance is needed for six basic functions (e.g., bathing and dressing) and eight instrumental functions (e.g., telephoning and food preparation). Scores are binary (0 = independent, 1 = dependent), resulting in a potential total score between 0 and 15, with a higher score indicating a higher level of dependency [38].
- *Resource utilization.* Medical facility use and nursing home admission will be recorded from a week before baseline assessment and throughout the follow-up period (part of TOPIC-MDS). Use of services, such as home care, day care, and visits by case managers in the week before assessment will be recorded at baseline and at three and six months.
- *The Dementia Quality of Life Instrument (DQI).* This tool will be used to assess the quality of life of the people with dementia. It covers five health domains (i.e., memory, orientation, dependency, social activities, and mood); derived from the EQ-5D, it is effective at measuring quality of life in dementia of mild to moderate severity [39].
- *The Cohen-Mansfield Agitation Inventory-Community (CMAI-C) scale.* This is an informant-rated measure of the frequency of agitated behavior in people with dementia. Results are collected through interview with the caregiver and the score can range from 29 to 203, with higher scores indicating agitation that is more frequent [40].
- *The Geriatric Deterioration Scale (GDS).* This scale will be used to classify people with dementia based on the relative severity of their cognitive impairment and their functional status (range from 1 to 7), with higher scores indicating more severe dementia [41].

### Focus group analyses

At meetings after 3 and 6 months, caregivers will be asked which intervention sessions were most helpful and whether the information they received was applicable to the care they delivered in practice. Also, caregivers will be asked about what information or subjects they felt were missed in the intervention and to specify what sessions were not useful or could be omitted. In preparation for its implementation in clinical practice, participants will also be asked how much they would be prepared to pay for the training if it had not been subsidized.

### Statistical analysis

#### Sample size calculation

The sample size calculation was performed based on the results of the first Australian study [24]. In that study, 96 participant dyads were included, with 65 in immediate and waiting groups and 31 in a control group. Enrolling 144 couples, equally divided over intervention and control groups, should be sufficient to demonstrate a medium effect size (average 0.5) with a significance of 0.05 and a power of 0.8. The anticipated attrition rate is 10%.

#### Outcome analysis

For the primary outcome, the change in the CarerQol-7D score from baseline to 3 months, a linear regression model will be developed to test the response variable for differences between the intervention and control groups, adjusting for the baseline outcome scores. We will perform a primary intention-to-treat analysis for the primary equation, which will be complemented with a secondary per-protocol analysis. The intention-to-treat population will include all information from participants randomized to each treatment arm, whereas the per-protocol population will only include information about those who completed the program to which they were allocated.

For the secondary outcome variables, multilevel analyses will be used to account for the dependency of the repeated measurements. A restricted, iterative, generalized least-squares algorithm will be used to estimate the regression coefficients, while the Wald test will be used to obtain *P* values for each regression coefficient. Linear or binary multilevel analysis will be conducted depending on the outcome measure. Subgroup analyses will be performed to assess the heterogeneity of treatment effects.

Assumptions of the normality and homogeneity of variance in the linear regression models will be assessed by inspecting normal probability plots and plots of standardized residuals compared with predicted values. If assumptions are not met, variables will be transformed to comply with the assumptions. If this does not yield acceptable distributions, variables will be dichotomized or we will use non-parametric statistical analyses. All statistical tests will be two-sided and will be required to meet the 5% significance criterion.

#### Missing data

We will conduct a missing value analysis to identify the patterns in missing variables, before determining whether the baseline characteristics differ between participants with and without missing data in terms of the response variables at the final measurement (6 months). Based on these analyses, we will infer whether missing values are likely to be missing at random (MAR), missing completely at random (MCAR), or missing not at random (MNAR). In the event of MCAR or MAR data, we will use multiple imputation techniques and present the outcome of analyses with and without the imputed data. In the event of MNAR data, we will not replace missing values and we will conduct case analysis when available.

### Ethics

The study has been submitted for approval to the Human Research Ethics Committee of the University of Groningen, the Netherlands. It was concluded that no assessment was needed based on the relevant law concerning scientific research in humans. However, the study will be conducted in accordance with the ethical standards of the 1964 Declaration of Helsinki (and subsequent revisions) and written informed consent will be obtained from all participating caregivers, and if possible, from the people with dementia.

## Discussion

More at Home with Dementia (*Beter Thuis met Dementie*) is a caregiver training program that we have adapted from a successful residential care setting in Australia and that will be delivered at a holiday location in the Netherlands. The presented study aims to determine the effectiveness of this intervention, primarily for the caregiver’s quality of life, after adapting it to the peculiarities of the Dutch health care system and to delivery in informal relaxed settings. This contrasts significantly with the approach used in the Australian studies where the intervention was delivered in nursing home or hospital settings. Standardized measures will be used to assess health-related primary and secondary outcomes among both the caregivers and the people with dementia. Additionally, we will use the opportunity to obtain qualitative outcome data from caregivers through focus groups. Ultimately, if the study outcomes confirm the intervention’s effectiveness, we anticipate that the results can be used to inform the implementation of this training program in routine care pathways. Using both the quantitative and qualitative results, we will also explore the generic aspects of the intervention that are not specific to dementia. For example, it is conceivable that the training content may apply to other conditions, such as Parkinson’s disease and multiple sclerosis, and for caregivers who do not live with the patient (e.g., children, other close relatives, or friends). Given the demographic changes in society, the growing burden of dementia, and the importance of care at home, we contend that intervention strategies should target not only the patient but also the informal caregiver. Therefore, this study will be of importance to both science and wider society.

## Abbreviations

ADL: Activities of Daily Living
CarerQol-7D: Care-Related Quality of Life-7 dimensions
CES-D: Center of Epidemiologic Studies-Depression
CMAI-C: Cohen-Mansfield Agitation Inventory-Community
DQI: Dementia Quality of Life Instrument
DSM: Diagnostic and Statistical Manual of Mental Disorders
EQ-5D + C: EuroQol-5 Dimensions + Cognition
GDS: Geriatric Deterioration Scale
GP: General Practitioner
HADS-A: Hospital Anxiety and Depression Scale, anxiety subscale
IADL: Instrumental Activities of Daily Living
M(C)AR: Missing completely at random
MAR: Missing at random
MNAR: Missing not at random
RAND-36/SF-36: RAND-36/short form 36
TOPICS-MDS: The Older Persons and Informal Caregivers Survey Minimum DataSet
VAS: Visual analog scale
